# Supplementary material for: High-Throughput Screening Assay for Detecting Drug-Induced Changes in Synchronized Neuronal Oscillations and Potential Seizure Risk Based on Ca2+ Fluorescence Measurements in Human Induced Pluripotent Stem Cell (hiPSC)-Derived Neuronal 2D and 3D Cultures
Source: Cells. 2023 Mar 21;12(6):958. doi: 10.3390/cells12060958 (PMC10046961; doi:10.3390/cells12060958)
Supplement: Supplementary file 1 [file cells-12-00958-s001.zip › cells-2229449-supplementary.pdf]

Table S1. Average and standard deviation of the effect of 25 reference drugs on the area under the curve of Ca^{2+} transients in hiPSC- derived neurons co-cultured with human primary astrocytes in 2D and 3D, (expressed as % change from baseline).

		Area under the curve - 2D								Area under the curve - 3D							
**Compound**	**Dose (μM)**	**Dose 1**		**Dose 2**		**Dose 3**		**Dose 4**		**Dose 1**		**Dose 2**		**Dose 3**		**Dose 4**	
		Mean	SD	Mean	SD	Mean	SD	Mean	SD	Mean	SD	Mean	SD	Mean	SD	Mean	SD
4-AP	0.37, 1.11, 3.33, 10	39.5*	13.2	18.7	13.0	17.6	12.3	1.3*	15.9	53.7*	24.5	54.1*	16.1	97.2	40.6	72.3	24.3
Acetylcholine	11.11, 33.33, 100, 300	38.3	34.6	19.9	26.2	−1.3	48.3	−6.1	24.8	109.9*	12.9	110.9*	20.5	97.3*	16.1	72.7*	89.1
Amoxapine	0.11, 0.33, 1, 3	27.8	23.3	25.3	11.4	22.8	6.9	−40.8**	15.3	122.7	33.8	130.2	28.7	124.0	26.1	148.1	42.6
Amoxicillin	0.11, 0.33, 1, 3	38.3	41.6	23.7	13.1	45.8	14.7	43.0	14.7	147.7	21.2	145.7	30.2	122.0	15.2	125.7	8.9
Aspirin	3.7, 11.11, 33.33, 100	48.5	19.5	25.7	25.5	37.6	23.5	30.0	18.7	139.0	14.0	139.2	14.4	147.8	19.8	133.2	10.8
Bicuculine	0.37, 1.11, 3.33, 10	15.8	40.4	39.6*	21.1	1.6	46.8	22.8	24.9	105.2	32.2	108.3	11.9	107.4	18.5	115.2	44.4
Bupropion	0.37, 1.11, 3.33, 10	14.5	15.1	26.6	16.6	25.5	20.4	39.3*	17.3	137.5	41.5	159.9**	27.1	154.1**	38.3	119.7**	15.6
Carbamazepine	3.7, 11.11, 33.33, 100	31.5	24.1	62.0	35.8	−39.7	66.8	−100.0**	0.0	122.1	7.6	121.7	13.5	107.4	15.0	173.0	56.0
Chlorpromazine	0.11, 0.33, 1, 3	26.5	7.7	36.0	24.0	10.7	7.6	−100.0**	0.0	128.5*	28.9	129.9**	16.1	132.1*	23.7	131.9**	15.5
Clozapine	0.04, 0.11, 0.33, 1	28.5	12.0	49.0*	17.4	28.8	34.9	10.8*	11.4	150.1**	34.9	153.9**	33.7	163.3**	14.3	182.8**	23.5
Cyclothiazide	3.7, 11.11, 33.33, 100	48.0*	15.5	18.7	19.8	−9.3	42.9	−53.8	71.6	135.8	24.8	187.3	13.0	245.1*	88.8	204.6**	89.9
Enoxacin	0.37, 1.11, 3.33, 10	25.5	22.4	41.0	20.9	47.3	19.9	49.3*	36.1	115.6	18.4	124.6	11.2	139.4	20.5	114.6	23.3
GABA	0.11, 0.33, 1, 3	23.1	18.2	21.1	19.1	16.4	41.6	14.9	42.4	111.5	21.7	116.2	17.4	97.0	37.0	118.7	8.0
Glutamate	1.11, 3.33, 10, 30	5.8	55.1	−13.0	52.2	−72.1*	27.0	−100.0*	0.0	116.3	40.3	129.9	26.3	128.0	42.4	116.3	20.1
Kainic Acid	0.37, 1.11, 3.33, 10	32.3	25.7	25.2	23.1	−48.2	41.4	−100.0	0.0	120.5	30.6	141.9	32.0	174.3	79.8	58.3**	34.8
Maprotiline	0.37, 1.11, 3.33, 10	12.2	14.8	−55.2**	9.7	−100.0**	0.0	−100.0**	0.0	139.7**	33.0	133.4**	23.7	123.1**	9.9	11.9**	24.7
Mefloquine	1.11, 3.33, 10, 30	14.8	26.4	−47.2**	5.9	−100.0**	0.0	−100.0**	0.0	159.0	8.2	143.4	9.3	135.6	21.2	74.4*	26.3
MK-801	0.11, 0.33, 1, 3	−35.7*	42.5	−57.0*	45.8	−57.4**	45.0	−79.8**	36.4	127.3	51.8	66.6	86.3	-66.9**	81.0	41.0**	79.8
Paracetamol (acetaminophen)	1.11, 3.33, 10, 30	23.0	25.2	35.8	27.7	13.2	26.9	−93.2**	16.7	104.3	22.6	121.7	30.6	122.4	7.5	132.0	12.8
Pazopanib	0.37, 1.11, 3.33, 10	−1.8	57.4	7.8	10.8	38.8*	19.1	16.3	23.1	125.7	53.1	127.9	25.1	116.3	16.9	117.4	11.0
Phenytoin	3.7, 11.11, 33.33, 100	33.2	17.2	27.5	8.3	15.8	14.6	33.3	8.3	132.1**	26.1	177.0**	32.2	199.3**	79.5	198.2**	34.5

Pilocarpine	0.37, 1.11, 3.33, 10	26.7	5.9	38.8*	15.0	26.7	28.6	22.8	12.6	141.5	61.4	173.5**	38.8	168.7**	14.1	145.2**	21.1
Quinacrine	0.37, 1.11, 3.33, 10	5.3*	13.9	11.2*	6.1	−13.8**	6.9	−84.7**	23.9	123.5	42.8	149.1*	45.6	139.1*	43.9	128.2*	13.8
Serotonin	3.7, 11.11, 33.33, 100	12.8	16.1	11.1	47.3	−8.9	50.2	−2.6	23.7	−19.4**	74.6	19.8**	66.4	−21.3**	66.6	−21.1**	79.1
Theophylline	11.11, 33.33, 100, 300	32.3	22.0	41.7	16.1	29.7	11.0	72.8**	24.8	156.1	23.7	171.1	49.0	148.8	78.5	134.2	82.1

*: p <0.05 vs. 0.1% DMSO, **: p <0.01 vs 0.1% DMSO.

Table S2. Average and standard deviation of the effect of 25 reference drugs on the peak-to-peak interval of Ca^{2+} transients in hiPSC- derived neurons co-cultured with human primary astrocytes in 2D and 3D, (expressed as % change from baseline).

		**Peak-to-peak interval - 2D**								**Peak-to-peak interval - 3D**							
**Compound**	**Dose (μM)**	**Dose 1**		**Dose 2**		**Dose 3**		**Dose 4**		**Dose 1**		**Dose 2**		**Dose 3**		**Dose 4**	
		Mean	SD	Mean	SD	Mean	SD	Mean	SD	Mean	SD	Mean	SD	Mean	SD	Mean	SD
4-AP	0.37, 1.11, 3.33, 10	−13.0*	14.6	−29.8**	13.3	−42.4**	22.1	−49.8**	12.0	−9.2**	11.3	−24.1**	25.0	−17.6**	15.1	−47.2**	7.5
Acetylcholine	11.11, 33.33, 100, 300	28.0	82.6	−25.8	76.1	11.3**	107.6	45.5*	160.3	89.6	98.3	38.9	69.0	−5.5	80.4	75.9	119.7
Amoxapine	0.11, 0.33, 1, 3	8.8	15.6	−7.8	37.0	−4.5	32.6	56.7	35.3	14.8	19.3	7.1	12.7	4.4	22.8	−6.6**	36.5
Amoxicillin	0.11, 0.33, 1, 3	27.3	17.9	9.3	66.0	−16.0	10.4	−7.8	24.9	12.1	15.8	9.0	14.9	−5.5	14.4	−1.2	15.1
Aspirin	3.7, 11.11, 33.33, 100	15.5	27.2	1.8	18.8	−0.5	27.7	−17.0*	20.5	10.9	20.8	−2.2	15.5	−1.7	16.9	6.7	20.2
Bicuculine	0.37, 1.11, 3.33, 10	−17.2	57.9	8.2	46.9	−26.1	42.0	−13.2	47.6	7.6	42.2	20.3	31.8	12.1	23.2	9.4	27.9
Bupropion	0.37, 1.11, 3.33, 10	−5.5*	14.4	3.4	26.2	19.7	23.3	41.8	96.0	4.1	19.2	15.3	18.8	7.8	15.4	−3.5	16.9
Carbamazepine	3.7, 11.11, 33.33, 100	0.8	61.0	45.8	43.9	45.0	170.1	−100.0**	0.0	−4.9	14.5	9.5	31.1	33.5	49.4	25.1	60.0
Chlorpromazine	0.11, 0.33, 1, 3	47.2	45.6	48.0	16.9	151.3**	79.9	−100.0**	0.0	10.0	38.8	12.5	35.4	−9.6**	17.5	6.0	15.0
Clozapine	0.04, 0.11, 0.33, 1	1.2	17.5	−15.5*	20.0	82.0	93.1	2.7	65.1	4.5	22.2	19.2	18.9	5.6*	10.3	14.2	14.9
Cyclothiazide	3.7, 11.11, 33.33, 100	9.2	29.7	−53.3**	12.4	0.3	78.5	−70.3	46.0	6.7	20.0	−14.5*	23.0	−30.0*	27.3	−49.3**	5.8
Enoxacin	0.37, 1.11, 3.33, 10	18.0	31.2	1.3	31.4	12.7	16.6	−3.0	30.4	30.9	33.3	−0.7	13.5	−1.3	21.4	−1.1	26.5
GABA	0.11, 0.33, 1, 3	−12.3	36.2	29.1	42.6	32.0	121.8	−26.3*	72.3	0.2	21.6	−2.1	18.1	8.3	35.1	−2.5	9.8
Glutamate	1.11, 3.33, 10, 30	−2.2	74.3	−17.4	62.7	96.3	372.1	−100.0	0.0	11.6	24.2	3.1	32.4	0.4	30.3	−1.6	19.4
Kainic Acid	0.37, 1.11, 3.33, 10	−1.2	35.3	−19.6	13.9	−53.0	59.6	−100.0	0.0	0.2	27.7	−16.0**	11.4	−13.0*	17.7	−45.2**	16.3
Maprotiline	0.37, 1.11, 3.33, 10	1.8	17.9	11.7	60.7	−100.0**	0.0	−100.0**	0.0	6.2*	14.8	8.4	17.2	10.6	17.7	−4.1*	19.9
Mefloquine	1.11, 3.33, 10, 30	25.0	40.1	48.2	135.5	−100.0**	0.0	−100.0**	0.0	7.1	14.6	1.7	17.1	12.8	51.3	8.3	42.5
MK-801	0.11, 0.33, 1, 3	203.5	254.8	3.6	231.7	−57.4	95.3	−100.0**	0.0	76.1*	28.7	66.4	149.3	−100.0**	0.0	−100.0**	0.0
Paracetamol (acetaminophen)	1.11, 3.33, 10, 30	34.0	23.1	−35.3	53.4	−14.5	116.9	−100.0**	0.0	12.7	19.3	17.4	29.6	−6.2	20.6	−8.8	21.6
Pazopanib	0.37, 1.11, 3.33, 10	14.6	67.0	−2.6	23.6	0.5	44.8	14.2	46.9	−2.0	32.5	4.1	7.0	−3.0	19.7	7.5	31.1
Phenytoin	3.7, 11.11, 33.33, 100	46.3	19.7	22.2	24.8	89.0*	50.4	44.5	33.9	2.9*	9.0	8.2*	12.1	17.7	19.8	6.6	13.1
Pilocarpine	0.37, 1.11, 3.33, 10	1.3	15.9	5.8	25.7	−9.2	18.3	29.8	28.6	−10.1	25.7	−10.0	27.7	−3.3	25.7	−12.6*	20.5
Quinacrine	0.37, 1.11, 3.33, 10	7.0	24.1	−4.0	8.6	40.2	85.8	−18.2	127.4	−0.7	20.4	6.2	27.5	−5.5*	15.5	−9.8**	10.7
Serotonin	3.7, 11.11, 33.33, 100	28.4	57.6	65.8	230.8	36.4	99.7	55.3	106.3	−58.0	62.7	−37.9	105.0	29.2	205.3	−82.0**	59.8

Theophylline	11.11, 33.33, 100, 300	24.3	22.5	−5.2	27.0	−26.3*	11.3	−46.8**	12.4	−4.0*	12.0	−21.2*	23.5	−37.1**	22.7	−45.7**	21.9

*: p <0.05 vs. 0.1% DMSO, **: p <0.01 vs 0.1% DMSO.

Table S3. Average and standard deviation of the effect of 25 reference drugs on the peak width of Ca^{2+} transients in hiPSC- derived neurons co-cultured with human primary astrocytes in 2D and 3D, (expressed as % change from baseline).

		**Peak width - 2D**								**Peak width - 3D**							
**Compound**	**Dose (µM)**	**Dose 1**		**Dose 2**		**Dose 3**		**Dose 4**		**Dose 1**		**Dose 2**		**Dose 3**		**Dose 4**	
		Mean	SD	Mean	SD	Mean	SD	Mean	SD	Mean	SD	Mean	SD	Mean	SD	Mean	SD
4-AP	0.37, 1.11, 3.33, 10	34.0	16.5	14.3	13.2	12.6	15.5	−6.3**	15.6	2.8	8.7	7.7	3.2	35.1*	19.3	21.4	14.3
Acetylcholine	11.11, 33.33, 100, 300	26.2	32.0	16.2	23.3	−3.8	47.1	2.6	29.8	4.2	6.0	0.4	4.8	0.0	7.2	−12.2	44.4
Amoxapine	0.11, 0.33, 1, 3	22.0	22.8	14.8	10.7	31.7	13.6	−37.7**	12.8	11.9	8.1	12.5	14.5	6.8	6.8	21.0*	18.5
Amoxicillin	0.11, 0.33, 1, 3	22.3	49.7	25.7	14.0	48.0	17.4	47.7*	17.6	4.7	4.2	5.5	10.9	−4.5	7.9	1.2	3.1
Aspirin	3.7, 11.11, 33.33, 100	43.6	22.5	25.9	26.0	38.3	25.2	32.1	24.6	4.2	2.9	0.3	2.5	5.0	9.9	4.7	7.7
Bicuculine	0.37, 1.11, 3.33, 10	16.1	41.6	33.0	18.2	3.7	48.7	11.5	21.8	−0.3	12.4	5.7	3.8	2.7	6.4	8.5	23.9
Bupropion	0.37, 1.11, 3.33, 10	12.8	10.4	26.6	21.2	29.2	20.8	46.5*	23.4	0.0	15.2	9.4	9.2	9.0	6.4	−1.4*	10.6
Carbamazepine	3.7, 11.11, 33.33, 100	19.0	28.0	66.5	45.3	−36.7	69.5	−100.0**	0.0	2.9	4.7	2.8	3.4	5.1	7.4	43.8**	28.8
Chlorpromazine	0.11, 0.33, 1, 3	18.0	8.5	38.5	31.2	13.8	7.0	−100.0**	0.0	13.3	10.0	13.6	11.2	13.0*	11.1	16.6	3.9
Clozapine	0.04, 0.11, 0.33, 1	20.8	19.1	35.5	16.6	22.8	30.9	11.0	15.5	6.2	9.2	8.6	10.3	14.0	4.5	22.6**	7.7
Cyclothiazide	3.7, 11.11, 33.33, 100	50.8	31.8	24.5	26.9	207.5	381.1	−17.3	128.1	0.7	7.7	23.6**	13.1	64.7**	39.4	80.0**	39.9
Enoxacin	0.37, 1.11, 3.33, 10	18.3	19.5	45.8	17.6	39.0	26.7	49.0	40.0	3.2	3.3	−2.2	9.7	3.0	3.8	−1.1	6.1
GABA	0.11, 0.33, 1, 3	13.2	21.4	15.4	19.9	11.7	39.7	8.5	38.5	7.6	6.9	0.5	1.7	−2.7	15.4	0.7	3.3
Glutamate	1.11, 3.33, 10, 30	−3.4	49.8	−5.2	57.0	−67.3*	31.5	−100.0*	0.0	12.5	11.3	8.6**	5.7	8.1	12.8	2.8	7.2
Kainic Acid	0.37, 1.11, 3.33, 10	22.0	31.4	37.4	16.2	−23.0	62.7	−100.0**	0.0	4.9	10.3	10.6	13.6	41.9	35.3	−20.1	24.0
Maprotiline	0.37, 1.11, 3.33, 10	4.5*	13.1	−57.2**	10.5	−100.0**	0.0	−100.0**	0.0	6.2	8.8	−1.0*	9.7	−3.2*	6.1	−40.8**	11.9
Mefloquine	1.11, 3.33, 10, 30	15.8	29.1	−47.0**	5.1	−100.0**	0.0	−100.0**	0.0	2.6	3.3	1.4	3.5	−1.5	7.1	−10.9	9.0
MK-801	0.11, 0.33, 1, 3	−3.2	66.1	−23.6	88.7	−32.8	77.7	−70.0	57.7	8.0	15.1	−29.3*	36.5	−85.8**	34.7	−36.8**	35.5
Paracetamol (acetaminophen)	1.11, 3.33, 10, 30	13.0	11.2	34.3	33.6	16.2	32.4	−92.5**	18.4	0.6	6.3	5.9	14.0	6.3	4.4	9.0	5.5
Pazopanib	0.37, 1.11, 3.33, 10	−4.8	58.2	12.6	17.8	27.8*	13.3	3.7	17.9	9.7	17.4	7.7	8.3	4.1	8.0	8.7	5.2
Phenytoin	3.7, 11.11, 33.33, 100	27.2	16.2	31.5	11.2	17.0	15.1	34.7	10.1	7.4	4.8	29.4*	13.8	47.9	35.6	50.5**	28.3
Pilocarpine	0.37, 1.11, 3.33, 10	19.2	7.4	35.7*	11.2	23.2	24.7	23.0	15.0	6.3	5.2	6.7	3.9	10.2	2.4	7.0	6.0
Quinacrine	0.37, 1.11, 3.33, 10	2.8*	12.9	10.7*	7.0	−12.8**	9.5	−84.3**	24.4	−1.8*	16.8	4.2	12.7	10.6	18.7	9.5	6.9
Serotonin	3.7, 11.11, 33.33, 100	17.3	17.5	18.3*	49.0	−1.4	53.3	11.4	23.8	−54.0**	41.8	−31.8**	36.5	−52.3**	40.4	−55.4**	42.8
Theophylline	11.11, 33.33, 100, 300	22.8	25.6	31.2	17.0	24.3	10.8	112.8**	49.2	11.1*	3.8	12.2*	10.0	21.3	36.8	29.3	57.6

*: p <0.05 vs. 0.1% DMSO, **: p <0.01 vs 0.1% DMSO.

Table S4. Average and standard deviation of the effect of 25 reference drugs on the peak amplitude of Ca^{2+} transients in hiPSC-derived neurons co-cultured with human primary astrocytes in 2D and 3D, (expressed as % change from baseline).

		**Peak amplitude - 2D**								**Peak amplitude - 3D**							
**Compound**	**Dose (μM)**	**Dose 1**		**Dose 2**		**Dose 3**		**Dose 4**		**Dose 1**		**Dose 2**		**Dose 3**		**Dose 4**	
		Mean	SD	Mean	SD	Mean	SD	Mean	SD	Mean	SD	Mean	SD	Mean	SD	Mean	SD
4-AP	0.37, 1.11, 3.33, 10	13.3**	2.3	7.8	7.4	10.4*	3.7	8.5	7.3	44.3	11.8	39.6*	9.5	46.1	11.5	42.8	13.3
Acetylcholine	11.11, 33.33, 100, 300	11.4	8.3	2.3	12.3	−17.0	39.3	−12.8	12.0	90.9	9.7	105.2	13.6	88.6	9.3	56.5	77.8
Amoxapine	0.11, 0.33, 1, 3	7.7	6.0	12.7	7.9	−1.5	5.3	−20.7*	11.0	92.4*	26.3	101.9**	18.2	102.7*	25.0	105.1**	19.2
Amoxicillin	0.11, 0.33, 1, 3	19.3**	3.0	5.0	2.6	8.3	3.6	7.5	4.3	136.9	21.8	132.0	18.7	130.1	14.4	123.5	7.3
Aspirin	3.7, 11.11, 33.33, 100	13.1*	6.5	4.2	7.3	6.2*	4.5	3.9	5.4	129.1	11.3	138.1	16.4	135.8	14.8	123.5	13.1
Bicuculine	0.37, 1.11, 3.33, 10	−4.1	32.1	9.3*	13.4	−14.1	38.1	9.8*	12.8	101.6	15.0	98.3	9.1	102.8	8.2	99.3	8.2
Bupropion	0.37, 1.11, 3.33, 10	2.7	7.6	5.4	3.6	3.7	3.3	4.7	4.7	126.7**	26.7	134.2**	13.9	128.1**	24.6	118.0**	13.7
Carbamazepine	3.7, 11.11, 33.33, 100	16.5**	6.3	8.5	5.8	−52.0**	52.7	−100.0**	0.0	114.1	11.1	114.1	14.1	93.5*	11.2	85.2	15.4
Chlorpromazine	0.11, 0.33, 1, 3	8.7*	3.4	4.0	2.8**	−4.3	3.5**	−100.0	0.0	96.7*	18.0	100.7**	10.8	102.4**	10.6	98.4**	12.1
Clozapine	0.04, 0.11, 0.33, 1	12.5**	4.9	16.7**	3.8	9.4*	7.6	1.0	2.9	133.6**	21.2	130.9**	14.9	129.8**	13.9	129.4**	14.0
Cyclothiazide	3.7, 11.11, 33.33, 100	12.3*	7.2	−0.5*	3.9	−24.0**	12.2	−72.8**	42.1	126.0	26.0	139.0	20.3	113.7	20.4	83.6*	26.3
Enoxacin	0.37, 1.11, 3.33, 10	7.8	9.8	5.3	5.6	13.5*	1.8	11.3*	5.7	106.8*	18.1	123.3	17.4	129.6	18.4	116.3	18.3
GABA	0.11, 0.33, 1, 3	9.1	6.9	5.7	5.8	−1.4	32.4	−2.7	32.9	87.8	24.3	112.0	15.1	90.5	22.1	110.9	9.1
Glutamate	1.11, 3.33, 10, 30	−8.5	45.5	−26.9	41.7	−60.9*	37.3	−100.0*	0.0	85.4*	28.8	108.3	20.3	103.9	23.6	107.0	15.0
Kainic Acid	0.37, 1.11, 3.33, 10	11.8*	4.3	−2.2	9.1	−55.0**	35.7	−100.0**	0.0	104.1	20.0	122.6	24.4	104.0	34.6	99.6*	8.7
Maprotiline	0.37, 1.11, 3.33, 10	8.3	5.6	−19.8**	4.2	−100.0**	0.0	−100.0**	0.0	116.3**	23.1	131.3**	10.8	130.1**	9.3	72.2	8.7
Mefloquine	1.11, 3.33, 10, 30	0.5	6.4	−21.2**	9.2	−100.0**	0.0	−100.0**	0.0	149.7	16.4	139.0	7.5	135.0	15.8	99.9*	18.2
MK-801	0.11, 0.33, 1, 3	−41.8**	29.8	−63.6**	34.2	−58.6**	38.3	−79.7**	31.6	104.7	20.9	76.2	89.3	−64.1*	88.0	67.2	94.0
Paracetamol (acetaminophen)	1.11, 3.33, 10, 30	8.8	16.3	12.3	11.1	−0.3	4.7	−89.8**	24.9	97.4	21.1	106.4	8.0	108.6	7.8	113.9	11.3
Pazopanib	0.37, 1.11, 3.33, 10	−15.8	47.1	−3.8	4.8	11.2*	7.2	9.5	14.3	101.6	21.8	110.2	10.6	107.5	4.5	101.7	6.1
Phenytoin	3.7, 11.11, 33.33, 100	7.3	3.8	−0.2	4.7	−3.0*	5.5	0.2*	1.8	111.5**	21.0	110.4**	12.7	91.2*	9.8	88.4	19.3
Pilocarpine	0.37, 1.11, 3.33, 10	9.3	4.8	8.0	4.7	5.8	10.1	3.8	3.8	115.6	54.9	145.8**	35.4	135.3**	14.8	122.5**	17.2
Quinacrine	0.37, 1.11, 3.33, 10	1.8	5.6	1.2	3.8	−7.8**	4.8	−78.0**	34.2	113.4*	28.7	128.3**	28.6	108.9*	41.9	108.3**	19.8
Serotonin	3.7, 11.11, 33.33, 100	−4.9	8.4	−13.7	29.7	−27.1	39.0	−16.6	7.9	−4.3**	85.3	33.7	72.3	−3.1**	80.7	−10.3**	88.3
Theophylline	11.11, 33.33, 100, 300	12.7	4.9	15.3*	3.7	10.0**	3.1	6.0**	6.5	121.2	16.2	142.1	29.3	99.6*	21.9	89.4**	10.2

*: $p < 0.05$ vs. 0.1% DMSO, **: $p < 0.01$ vs 0.1% DMSO.
